# The specter of authenticity: Social science after the deconstruction of Romanticism

**DOI:** 10.1177/09526951241245014

**Published:** 2024-05-24

**Authors:** Galen Watts, Dick Houtman

**Affiliations:** 8430 University of Waterloo, Canada; 26657 KU Leuven, Belgium

**Keywords:** authenticity, critique, culture, Romanticism, theory

## Abstract

In a long-forgotten essay, Alvin Gouldner defended the distinctive contributions of Romantic social science. Today, half a century later, very few would risk making a similar plea. Owing to its deconstruction, the discourse of Romanticism has increasingly fallen out of favor in the social sciences, meaning social scientists have progressively come to see Romanticism as less a *resource* for critique than a bourgeois *ideology* warranting critical scrutiny. Yet the truth is quite a bit more complicated. For despite its disapproval at the level of social science's *explicit* culture, Romanticism continues to serve, at the level of *implicit* culture, as a potent resource for social analysis. We start with a clarification of what we mean by Romanticism. While Romanticism may be an amorphous and multifaceted structure of thought and feeling, like Gouldner, we do not think it lacks coherence. Thus, we outline what we take to be the core dimensions of the ‘Romantic syndrome’, and then survey some of its key figures in Western social thought. Next, we move to a discussion of three select studies about the infiltration of Romanticism into the capitalist heartland—the sphere of work. We demonstrate how, consistent with our argument that Romanticism has become increasingly symbolically polluted within social science, each of these studies critiques the Romantic turn at work, while nevertheless anchoring their critiques in Romanticism, albeit in increasingly implicit fashion. We conclude by offering some reflections on why Romanticism continues to haunt contemporary social science—and why this matters.

The ‘modern’ only begins to manifest itself, when in answer to the question, ‘What is distinctively human?’, Romanticism replies not by referring to man's eternal capacity for reason and universal rationality, but, instead, to his creative originality, to his individuated capacity to feel and dream uniquely. (Gouldner, 1973: 330)

A specter is haunting the human sciences—the specter of Romanticism. In a long-forgotten and much-neglected essay, Gouldner (1973: 358) sketches and juxtaposes Romanticism and classicism as ‘deep structures’ in social science. He argues that these two ‘syndromes’ embed distinct domain assumptions and animate specific theoretical infrastructures, while continuing to shape and suffuse social scientific analysis. Writing in the early 1970s, Gouldner maintained that Romanticism and classicism remained critical ‘intellectual tools for the empirical study of the ongoing social sciences today’ (ibid.: 358). Yet he was not simply interested in elucidating these contrasting structures of thought and feeling for the sake of increased theoretical clarity. Gouldner also sought to advance a (measured) defense of what he referred to as the ‘special value of the *Romantic* infrastructure’ (ibid.: 361). As anyone who has read Gouldner's classic *The Coming Crisis of Western Sociology* knows, he was a trenchant critic of Parsons's structural-functionalism, for he saw it as a paradigmatic example of classical sociology, chiefly concerned with assimilating individuals to their social roles and maintaining the stability of the social order. And given Parson's hegemony in American sociology at the time—what Gouldner referred to as an ‘establishment-sponsored Classicism’ (ibid.: 361)—he sought to defend the distinctive contributions of Romantic sociology.

In advancing his case for Romantic social science, Gouldner was channeling the cultural currents pulsing throughout the wider society of the 1960s. Let us not forget that ‘the spirit of May 1968’, as it is known in France, reflected no less than a Romantic revival, perhaps even revolution, of sorts (Bellah *et al.*, 1985; Campbell, 2007). Despite their claims to novelty, members of the ‘counterculture’ and its associated liberation movements (the New Left, feminism, gay liberation, pacifism, ecology, etc.) were, as Berman (1972) famously remarked, reviving the ‘politics of authenticity’ typical of Romantics before them. Gouldner was acutely aware of this. One of the first to champion a ‘reflexive sociology’, he was deeply sensitive to the dialogical and interdependent relationship between social science and the culture from which it spawns. His was therefore a self-conscious attempt to give systematic voice to the Romanticism animating the social movements of the era.

Today, half a century later, very few social scientists would risk making a similar plea. As Hagel (2017: 221) observes, ‘For many in the academy, appeals to authenticity have become suspect.’ Ironically, much of this skepticism is due to the rapid expansion of a ‘culture of authenticity’ (Taylor, 1991) since the 1970s, when the discourse of Romanticism began to make major inroads into the social spheres of work (Boltanski and Chiapello, 2005; Watts and Houtman, 2023a), consumption (Frank, 1998; Heath and Potter, 2004; O’Neill, Aupers, and Houtman, 2014), and religion (Campbell, 2007; Heelas and Woodhead, 2005; Watts, 2022)—all spheres critiqued by counterculturalists of the 1960s as standing in the way of personal authenticity and free expression. In response to these developments, a wave of deconstructive critiques were launched (e.g. Bourdieu, 1984; Foucault, 1978). These originated in France, but over the ensuing decades spread across Europe and North America, exerting a powerful influence. Accordingly, the dominant view in the social sciences today is that ‘authenticity talk’ in these spheres is basically a hoax, or evidence of new modes of capitalist interpellation and social control (e.g. Boltanski and Chiapello, 2005; Carrette and King, 2005; Goldman and Papson, 1996). Thus, in the wake of its ‘radical deconstruction’ (Boltanski and Chiapello, 2005: 451), the discourse of Romanticism has increasingly fallen out of favor, meaning social scientists have progressively come to see Romanticism as less a *resource* for critique (as Gouldner did) than a bourgeois *ideology* warranting critical scrutiny. Yet we hope to show in what follows that the truth is quite a bit more complicated. For despite its disapproval and disavowal at the level of social science's *explicit* culture (see Simko and Olick, 2021), Romanticism continues to serve, at the level of *implicit* culture, as a potent resource for social analysis.

Our argument proceeds as follows. We start with a clarification of what we mean by Romanticism. One reason social scientists may be skeptical of ‘Romanticism’ is because the term ostensibly lacks analytical bite, and perhaps even seems to ‘defy analysis’ (Löwy and Sayre, 2002: 1). While Romanticism is indeed an amorphous and multifaceted structure of thought and feeling, like Gouldner, we do not think it lacks coherence. Thus, we outline what we take to be the core dimensions of the ‘Romantic syndrome’, and then survey some of its key figures in Western social thought. Next, we move to a discussion of three select studies that take up the infiltration of Romanticism into the capitalist heartland—the sphere of work. We demonstrate how, consistent with our argument that Romanticism has become increasingly symbolically polluted within social science, each of these studies critiques the Romantic turn at work, while nevertheless anchoring their critiques in Romanticism, albeit in increasingly implicit fashion. We conclude by offering some reflections on why Romanticism, despite its limitations and deficiencies, continues to haunt contemporary social science.

This article seeks to make three contributions to contemporary social scientific debates. First, we hope our account of Romanticism may be used to guide future empirical studies. Not only does this cultural tradition retain an enduring appeal among social scientists, but it significantly structures contemporary culture and identity in Western societies (two facts that are, of course, not unrelated). Thus, consider this article our modest (if also bold) attempt to define and foster a sociology of Romanticism. Second, inspired by Gouldner, we wish to initiate a reflexive discussion about the relationship between social science and the wider culture in which it spawns and circulates. We agree with Gouldner (1970: 13) that ‘social science is a *part* of the social world as well as a *conception* of it’, and so think we social scientists would benefit from reflecting upon the ‘normative and existential assumptions’ (ibid.: 32) naturalized within our theories, and the role these play in securing appeal and acceptance from our readers. Third, if we accept Gouldner's claim that social scientific theories revive and renew enduring structures of thought and feeling, then it follows that social science *itself* is open to cultural analysis. The final contribution of this article, then, is to illuminate the subtle ways in which social scientific analysis—*as a form of culture*—actually operates.

## What is Romanticism?

In their penetrating analysis of the Romantic *Weltanschauung*, or worldview, Löwy and Sayre (2002: 28) contend that Romanticism remains ‘a veritable lost continent that has escaped the usual grids of the human sciences’. Part of the reason for this, they admit, is because of its ‘fabulously contradictory character’ (ibid.: 1). As we shall see, Romanticism has been (and continues to be) appropriated and adapted for all manner of ends. Yet it is also explained by the inability of social scientific categories to classify this meaning structure. As Löwy and Sayre (ibid.: 27–8) observe, ‘Since it does not correspond to the usual categories (in philosophy, rationalism, empiricism, idealism; in history and politics, left-right, conservatives-liberals, progressives-reactionaries), it slips through the social scientists’ nets and generally remains invisible in their analyses.’ Thus, they contend that social scientists remain wary of Romanticism because they lack the classification schemas to make sense of it. But what, then, does this category-defying structure consist of?

According to Isaiah Berlin (1999) it was in the 18th century that the Romantic creed was first articulated. Although many have traced the seed of Romanticism to Rousseau (see Babbitt, 1919), Berlin traces it back to Herder, for whom ‘the fundamental function of human beings was to express, to speak, and therefore that whatever a man did expressed his full nature and if it did not express his full nature, it was because he maimed himself, or restrained himself, or laid some kind of leash upon his energies’ (Berlin, 1999: 58). From this ‘expressive view of human life’, as Taylor (1989: 375) calls it, flow all of the other tenets of Romanticism. For the Romantic, the most horrifying fate is that of being *cut off from*, or *estranged* from, one's *authentic self* (Larmore, 1996). Indeed, as M. H. Abrams (1973: 182) observes in his classic study, Romantics insist upon a ‘metaphysics of integration’; the Romantic longs for a world where they can feel *at home*, and *at one* with themselves and the world. *Reunification*, *unity*, *totality*—these are the watchwords of the Romantic. Further, Romantics are obsessed with *freedom* and *liberation*—yet for them, these ideals speak primarily of the inner life. As Löwy and Sayre (2002: 25) observe, for the Romantics there is nothing more sacred than the ‘subjectivity of the individual, the development of the richness of the human personality, in its full affective depth and complexity, but also in the full freedom of its imaginary’. *To realize oneself in one's fullness*—this is the telos of Romanticism. Nevertheless, counter to common misconceptions, this need not entail a rugged individualism. No doubt, Romanticism can fuel an antinomianism that delights only in the peculiar, the odd, and the nonconformist (Berlin, 1999: 65). Yet the Romantic longing for inner unity and freedom can just as well fuel a nostalgic longing for ‘authentic community’ (Löwy and Sayre, 2002: 42). In fact, Romantics are prone to a heady nostalgia for a fictionalized past, where humans were allegedly freer, more whole, and less cut off from the natural world. Thus, Rousseau was clearly a Romantic of sorts; his ‘Noble Savage’ is nothing but a quixotic fiction—a product of that melancholic nostalgia so typical of the Romantic soul.

Further, it is owing to this longing for a ‘paradise lost’ (Sayre and Löwy, 1984: 57), which is then projected onto the future, that Romanticism tends to manifest as ‘restlessness, questioning, perpetual becoming, searching, struggle’. The Romantic therefore tends toward a perpetual dissatisfaction with the historical present, as their attention is always oriented to both a *fictionalized past* and a *utopian future*. Here, then, lie the seeds of what Gouldner (1973: 332) called the ‘emancipatory standpoint’ within this deep structure: at the core of Romanticism lies a trenchant suspicion of existing institutions and social forms. For the Romantic, evil does not originate in the human heart, but rather is planted there by society. Or, to paraphrase Rousseau: we are born free, but everywhere in chains. The implications of this conviction are many, but as regards styles of social scientific thought, this explains why, at the center of Romantic social science lies the assumption ‘that men's [*sic*] humanness is limited by the established society’ (Gouldner, 1973: 360).

Romanticism emerged in the 18th century as a critique of the rationalism and materialism embodied in the Enlightenment (Brunkhorst, 1987). The Romantics were artists, poets, writers, theologians, and philosophers who shared a deep concern regarding the fate of subjectivity, value, and mystery in modernity. To use a Romanticism-tinged term later made famous by Weber, they feared a ‘disenchantment of the world’, wrought by industrial capitalism, technological advancement, and scientific materialism. Thus, it is only *as a critical response* to these perceived threats that we can make sense of the distinctive character of Romanticism as a structure of feeling and thought. Dreading the loss of transcendental meaning in modernity, the Romantics sought to re-enchant the world through imagination, art, and reconnecting with the natural world (Abrams, 1973). From Novalis, to Coleridge, to Schelling, the value of art lay in its ability to reinfuse the mystical and magical back into a reified and mechanistic world. Horrified by the moral and epistemological effects of capitalist, bureaucratic, and industrial processes of rationalization and standardization upon the human spirit, the Romantics championed subjectivity, personal experience, raw feeling, imagination, and the ‘creative originality’ of individuals (Gouldner, 1973: 331). And aghast at the loss of nonpecuniary values as the money economy expanded its reach, Romantics championed, in democratic fashion, the infinite value of the mundane, of the simple, and even of *life* itself. As Löwy and Sayre (2002: 42) summarize, the Romantics were ‘painfully aware of the alienation of human relationships, the destruction of the old organic and communitarian forms of social life, the isolation of individuals in their egoistic selves … at the very center of modern social life’.

## Romanticism in Western social thought

Having sketched its basic infrastructure in broad form, it should now be clear why Gouldner (1973: 324) saw Romanticism as a ‘powerful movement for cultural revitalization’ that has long animated Western social theory. Indeed, it is near impossible to conceive of much social thought without the contributions afforded by what Hansen (1997) refers to as the ‘romanticist episteme’.

Consider, first, the Romantic aspects of Marx's thought. Although it is rarely noted today, Marx's notion of ‘alienation’, central to his early work, is profoundly Romantic in both content and form (Abrams, 1973: 316; Breines, 1977: 473; Gouldner, 1973: 337; Löwy and Sayre, 2002: 96; Trilling, 1972: 124). For the young Marx—who in his adolescence dreamed of becoming a poet and playwright (Breckman, 2022)—what defines humans is their creative capacities (their ‘species being’), which capitalist relations of production stifle and subdue. Owing to the nature of work under capitalism, the worker is estranged from his (for Marx assumed the worker was male) labor power, and thus, one might say, his authentic self (Abrams, 1973: 316). For Marx, then, ‘alienation’ captures a characteristically Romantic concern about self-estrangement. But this is not all. As Gouldner remarks, when Marx speaks of the ‘contradictions’ of capitalism, he is implicitly drawing upon Romantic ideals of integration and unity; what makes capitalism grotesque is precisely its fragmenting and splitting effect on the human personality (Gouldner, 1973: 339). Furthermore, it goes without saying that Marx's critique of the ‘cash nexus’ derives much of its normative thrust from Romantic ideals of subjectivity, depth, and authenticity; the problem with money is that it corrupts social relations, destroys traditional practices, and empties the world of transcendent meaning and value (Berman, 1972). Moreover, a money economy, fueled by the drive for efficiency and profit, inevitably leads to a dehumanizing standardization, which crushes any room for individual or collective self-expression. Thus, as Trilling (1972: 124) remarks, for Marx, ‘money, in short, is the principle of the inauthentic in human existence’. Last but not least, it is not difficult to find in Marx's early work a nostalgic utopianism—with its heady mix of pastoral fantasy and technological optimism—which bears uncanny resemblances to the communal ideal that Berman (1972: ix) views as central to the politics of authenticity—that is, a world where individuality blossoms, yet where social relations remain deep and harmonious.

Of course, this is not to suggest that Marxism is tantamount to Romanticism. It is obviously the case that Marxism draws upon a variety of normative sources. Moreover, if we accept Gouldner's (1980) analysis, Marxism is best conceived as comprising two quite different intellectual traditions. The later ‘Scientific Marxism’, as Gouldner called it, has little in common with Romanticism, rooted as it is in a Classical paradigm and indebted to Enlightenment rationalism. For Scientific Marxists, Marxism is a *science* of society, focused on discovering the laws of history and social life. By contrast, the earlier ‘Critical Marxism’, which is less science than critique, owes much to Romanticism.^1^ As Gouldner explains, ‘The fundamental goals of Critical Marxism are to preserve human culture and certain transcendental values, to reinvigorate human morality, and to restore men to a life in an integral community’ (ibid.: 60). It would be difficult to summarize Romantic humanism any better than this. Thus, Critical Marxists are basically revolutionary Romantics, fundamentally concerned with the problems of alienation and inauthenticity, and the pernicious effects of capitalism on the human spirit.

Writing in 1980, well before the fall of the Berlin Wall, Gouldner described Scientific Marxism as the dominant form, but also noted that a small coterie of dissonant voices on the left were responsible for keeping Critical Marxism alive in the 20th century. Let us briefly note a few. If there was ever a paradigmatic representative of Critical Marxism it would have to be Herbert Marcuse. In *One-Dimensional Man* Marcuse proclaims: ‘To the degree to which consciousness is determined by the exigencies and interests of the established society, it is “unfree”; to the degree to which the established society is irrational, the consciousness becomes free for the higher historical rationality only in the struggle *against* the established society’ (Marcuse, 1964: 222; emphasis in original). In this text—which became a sort of manifesto for the New Left—we find all the basics of Romantic thought: despite their surface contentment, Marcuse maintains, the American middle classes are in fact suffering from a condition of deep alienation—the result of living in a society that superimposes upon them needs that are not authentically their own. In turn, Marcuse basically offers a ‘neo-Romantic mode of cultural criticism’, according to which ‘the priority really [is] to “to free one's mind”’ (Campbell, 2007: 294–5).

Of course, we could also include in this list other German theorists: Lukacs, Benjamin, and Fromm, who, as Breines (1977: 479; emphasis in original deleted) observes, in their own ways ‘sought to restore Marxism its lost Romantic dimension’. In fact, it would not be unreasonable to view the Frankfurt School more generally as indebted to Romanticism in one form or another. For instance, despite Adorno's (1973) trenchant criticism of the ‘jargon of authenticity’ it is not difficult to detect the Romantic episteme in his and Horkheimer's warnings of the rise of instrumental reason in modernity (Gouldner, 1973: 336). For recall: Weber's thesis of disenchantment, which Adorno and Horkheimer revive in their classic *Dialectic of Enlightenment*, can be traced back to German idealism—itself a product of Romantic infrastructure (Josephson-Storm, 2017).^2^ What unites these various critical theorists, and what places them within the Romantic tradition, Dant (2003: 158) explains, is their commitment to freedom understood not as ‘freedom from physical bondage or violent repression’, but rather as ‘freedom for individuals to live their lives to the full: freedom from alienation’.^3^ Yet it was not only German émigrés who were responsible for carrying forward the Romantic tradition of Critical Marxism. The UK has a long-standing tradition of Romantic socialism, whose representative figures include William Morris, Raymond Williams, and E. P. Thompson. In fact, according to Löwy and Sayre (2002: 54), in many ways these British New Leftists best capture the ‘Romantic anti-capitalist worldview’—or what Berman (1972: xi) felicitously refers to as ‘Marxism with soul’.

At the same time, Marxism is far from the only carrier of Romanticism in Western social thought. In France, it was the existentialists—particularly Sartre—who popularized and systematized the Romantic episteme. Indeed, it is arguably owing to Sartre's radical voluntarism that Romanticism developed a reputation for being *un*, if not thoroughly *anti*-, sociological (cf. Bourdieu and Wacquant, 1992). Yet, in truth, Romanticism has long infused classical social theory. For instance, many have highlighted the Romantic dimensions of Weber's thought (Watts and Houtman, 2023b). This may seem strange, given Weber's reputation as a sober-minded rationalist, whose doctrine of ‘value neutrality’ remains the aspirational ideal of positivists to this day. Yet, as Gouldner (1973: 342; emphasis added) perceptively notes, Weber's doctrine of value neutrality always presupposed a deeply Romantic worldview:

The creation of social science is, in Weber's view, seen as contingent ultimately on the exertion of essentially personal powers rather than professional skills. Its focal concern is on the quality of a man's [*sic*] inwardness, his sense of responsibility, individual intuition and empathy, rather than on the cumulative resources of the scientific community outside of himself. *The Weberian conception of social science thus entailed a systematic application of Romantic premises*.

And Romanticism's reach stretches further yet. Influenced by German idealism while studying in Berlin, W. E. B. Du Bois incorporated Romantic assumptions into his social thought in order to diagnose and critique the evils of segregation and systemic racism in America; one of the most insidious consequences of white supremacy, argued Du Bois, was that it prohibited African Americans from self-actualizing, leaving them in a perpetual state of division or ‘twoness’ (Itzigsohn and Brown, 2020). In *The Souls of Black Folk*, Du Bois (2007[1903]: 9) writes, ‘The history of the American Negro is the history of this strife,—this longing to attain self-conscious manhood, to merge his double self into a better and truer self.’ Finally, given his significant debts to Weber, it should not surprise us to find traces of Romanticism in the social thought of C. Wright Mills. In fact, Gouldner contends that ‘the main themes’ of Mills's classic *The Sociological Imagination* ‘represent an essentially Romantic perspective’ (1973: 355). Gouldner bases this claim, first, on the grounds that Mills rejects all impersonal methodologies—thereby stressing the subjective dimensions (intuition, empathy, insight) of good sociological practice—as well as his vehement hatred of ‘mindless statistical empiricism’ and ‘abstracted grand theorizing’ (ibid.: 354). However, Mills's Romanticism is arguably most apparent in his choice of title: it is no coincidence that he described it as the sociological *imagination*—the latter being a keyword of Romanticism (Larmore, 1996). Hence Gouldner's (1973: 355) conclusion that Mills's conception of sociology ‘was plainly Romantic’.

No doubt, many other prominent social theorists could be listed. But we think this list suffices to make apparent both the coherence and significance of this meaning structure in the history of Western social thought.

## The Romantic turn of the 1960s: From the cultural margins to the center

When Gouldner advanced his defense of Romantic social science in the early 1970s as a means of counterbalancing the ‘establishment-sponsored Classicism’ he observed in American sociology, he sought to channel the Romanticism emergent in the wider society. From second-wave feminism to the movements for gay liberation and black power, to the environmental movement, Romanticism was a critical resource in the fight against what counterculturalists disdainfully deemed the ‘system’ (Roszak, 1969). Espousing a politics of authenticity, feminists engaged in consciousness-raising in order to identify and uproot the cultural conditioning imposed upon them by a patriarchal society (Colker, 1988: 218). Similarly, both black and gay activists appealed to ideals of authenticity in order to challenge the discriminatory practices and prejudicial assumptions embedded in the homophobic and anti-black social climates of Western societies (Friedman, 1990). While environmentalists took aim at the reifying and destructive effects of industrial capitalism, whose rapacious extractivism, they argued, not only blinded moderns to the intrinsic value of the natural world, but also alienated them from this life-giving source (Watts, 2022).

What Gouldner likely could not have predicted was that these nascent cultural strands would, in a matter of decades, completely transform the cultural and institutional mainstream—becoming something of a ‘common sense’. Yet study after study demonstrates that this is the case. Romantic discourses of authenticity, autonomy, self-expression, creativity, passion, and liberation are now naturalized in institutional spheres as wide-ranging as the family (Bellah *et al.*, 1985; Furedi, 2004), politics (Fordahl, 2018; Fukuyama, 2018), law (Stolzenberg, 2009), religion (Bielo, 2011; Campbell, 2007; Watts and Houtman, 2023b), and consumption (Frank, 1998; Illouz, 1997; O’Neill, Aupers, and Houtman, 2014). Indeed, it is for precisely this reason that Taylor (1991) is correct to deem ours a ‘culture of authenticity’.

Yet what is most striking is the extent to which Romanticism has become a central feature of the contemporary sphere of work.^4^ Pointing out the increased salience of discourses of liberation, expression, and personal authenticity, Boltanski and Chiapello (2005) famously contend that Romanticism now informs what they call ‘the new spirit of capitalism’, which has made 21st-century capitalism profoundly Romantic (see also Heelas, 2008). Just a few examples should make this apparent: Google recently launched an employee training program titled ‘Search Inside Yourself’, which presents the Romantic quest of self-realization as complementary to the capitalist goal of increased productivity. Facebook now champions a ‘play ethic’, while boasting that its employees are free to be their ‘authentic selves’. *Forbes* magazine regularly features articles that present the pursuit of authenticity and profitability as twin features of any successful business plan. And the most popular self-help book for job seekers, Richard Bolles’s *What Color Is Your Parachute?* (1996), claims that the secret to professional success is achieving personal authenticity (see also De Keere, 2014).

Boltanski and Chiapello (2005) contend that this shift is best understood as capitalism's appropriation of the Romantic (what they call ‘artistic’) critiques of the 1960s, which charged capitalist corporations with producing worker alienation, repression, despair, and conformity. Management was quick to respond and adapt (Thrift, 1997: 49), sparking a major transformation in the dominant corporate ethos. Dress codes have been relaxed, self-expression encouraged, authenticity celebrated, and creativity prized over conformity. New buzzwords, drawn from Maslow's humanistic psychology and the budding field of happiness studies—‘emotional intelligence’, ‘self-actualization’, ‘wellness’—have become staples of neo-management discourse, as have new corporate policies that strive to integrate ‘work and play’ (Costea, Crump, and Amiridis, 2008: 675; see also 2007). These changes have been coterminous with a number of economic and technological changes—the shift to a knowledge economy; a simultaneous shrinking of manufacturing and growth of the service sector; a rise in outsourcing, automation, and contract work; and the normalization of insecurity and precariousness—which together mark a radical break with the employer-employee contract enshrined after the Second World War. These various changes have been subsumed under different labels—including ‘soft capitalism’ (Thrift, 1997), the ‘new economy’ (Löfgren, 2003), and ‘the new capitalism’ (Sennett, 2006). But what these disparate appellations share is a recognition that the late modern world of work is deeply infused with Romantic discourse (Anteby and Occhiuto, 2020; DePalma, 2021; Pagis, 2021).

## Explicit and implicit modes of Romantic social science: Three ideal types

In the 1970s and 1980s, Romantic discourse became the target of scathing intellectual critique. This academic trend began in France; faced with the bourgeois embrace of Romanticism, French critics such as Bourdieu and Foucault launched aggressive fronts against the ‘thematic of authenticity’, subjecting it to ‘a systematic labour of deconstruction’ (Boltanski and Chiapello, 2005: 453). For Bourdieu (1984), authenticity talk was merely a class-inflected attempt at *distinction*, a new means by which the upper classes legitimated their prejudices and preserved their power. For Foucault (1978), inspired by Nietzsche, claims of authenticity naively naturalized discourses whose ‘truth’ was not only constructed, but also contingent upon a normalizing power–knowledge nexus. Although it took some time, in the ensuing years, social theorists and cultural critics alike have largely incorporated the spirit, if not the substance, of these deconstructive critiques (e.g. Feldman, 2015; Potter, 2010). No doubt, the symbolic pollution attending Romantic discourse has much to do with its mainstreaming since the 1970s. It is arguably because of the remarkable success of capitalism's appropriation of the Romantic thematic that explicitly aligning oneself with Romanticism has become deeply taboo in the social sciences (cf. Hagel, 2017).

But does this mean that Romanticism no longer serves as a resource for social analysis? We think not. Rather, in the wake of Romanticism's deconstruction since the 1970s, many social scientists have continued to appropriate it, but in increasingly implicit forms. And to explain what we mean, we draw from recent innovations in cultural theory.

In an important article that aims to clarify the varieties of culture, Simko and Olick (2021) distinguish between *practical* and *discursive* culture. The former refers to embodied competences, dispositions, and types of memory, whereas the latter refer to articulable language, signs, and codes (see also Lizardo, 2017). Moreover, Simko and Olick contend that both types of culture contain *explicit* and *implicit* facets. In the case of discursive culture—the kind that interests us here—the explicit dimensions include ‘symbols, rituals, words, slogans, emblems, manifestos’, and the like, while the implicit dimensions can include ‘hidden codes, “deep” structures, archetypes, or systems of relations often underscored in studies of myth, genre, or metanarrative’ (Simko and Olick, 2021: 440). Expanding on this distinction, Simko and Olick write that ‘explicit-discursive culture’ ‘consists of the concrete discursive products of the human imagination’ (ibid.: 443), adding that ‘Stories, speeches, ceremonies, fashion, weddings, hats, slogans, symbols, and legitimacy claims are all discursive culture insofar as they express or represent shared ideas and meanings’ (ibid.: 443). By contrast, ‘implicit-discursive culture’ ‘refers to the group- or societal-level cultural codes that can be readily mobilized to construct ideas’ (ibid.: 443) but which remain inarticulate, unspoken, and perhaps even invisible. This analytic distinction is extremely useful for making sense of how Romanticism lives on in contemporary social science, for we maintain that although it is increasingly symbolically polluted at the level of *explicit* social scientific culture—such that very few social scientists are willing to unironically champion the language and ideals of Romanticism—this structure of thought and feeling nevertheless remains active and influential at the level of *implicit* social scientific culture—orienting empirical research, providing normative and existential assumptions, and animating common readings.

In the remainder of this article, we demonstrate this through a discussion of three select textual case studies. Our choice is informed by a logic of ‘humanist interpretation’, as defined by Biernacki (2014: 180), which follows Max Weber's methodology in not seeking ‘statistical averages’ but rather ‘iconic exemplars’ of distinct ‘ideal types’. In his trenchant (if somewhat polemical) critique of ‘coding’ in qualitative research, Biernacki (2012: 146) contends that because ‘meanings in operation remain tied to concrete prototypes’ coding leads to the reification of fictional abstractions, produced by the researcher, that have no correspondence in social reality. Because meanings are moreover ‘radically individual’ (ibid.: 178), it is a categorical error to seek ‘representativeness’ in the sense of ‘average’ or ‘representative’ meanings. Consider, for instance, how in *The Protestant Ethic and the Spirit of Capitalism* Weber (2005[1904/5]) did not describe ascetic Protestantism in reified abstraction—that is, as the statistical average of Puritan inner life—but instead offered Richard Baxter as an illustrative prototype. Needless to say, this choice follows logically from Weber's interpretive philosophy of social science.

Applying this logic, we chose three books: Arlie Hochschild's *The Managed Heart: Commercialization of Human Feeling* (1983), about the instrumentalization and commercialization of human emotions in postindustrial service work; Peter Fleming's *Authenticity and the Cultural Politics of Work* (2009), which critiques the corporate turn to authenticity as entailing counterfeit rather than genuine authenticity; and Ofer Sharone's *Flawed System/Flawed Self* (2013), which critically examines cultures of job seeking in America and Israel. We did not choose these books because we believe they are somehow ‘representative’ of contemporary social scientific culture writ large, but rather because we believe them to exemplify three different *ideal types*, which enable us to delineate three distinct ways Romanticism lives on in contemporary social science. Whereas each of the three selected texts has a distinct empirical and theoretical focus, all three take up (albeit in their own ways) the infiltration of Romantic discourse, motifs, and ideals into the world of work. Moreover, all seek to not only *describe* but also *normatively critique* what they observe—invoking a Romantic infrastructure in doing so. Finally, as they were written at different points over the last half-century, we believe their comparison usefully captures an important trend as it relates to the symbolic pollution of explicit Romanticism.

## Explicit Romanticism I, expressive individualist: Arlie Hochschild's *The Managed Heart*

The first study we consider is Arlie Hochschild's classic *The Managed Heart: Commercialization of Human Feeling*, published in 1983. This book was published not long after the first wave of deconstruction swept across the social sciences. Given this fact, one might reasonably expect it to be far more *explicitly anti-Romantic* than it is. The reason it is not, we suggest, arguably lies in the book's American provenance—the deconstructive currents pervading the French academy had not yet swept through North America in the early 1980s. So, while *The Managed Heart* certainly suggests a clear awareness of these trends—in dutiful sociological fashion, Hochschild makes sure to create an *ironic distance* between herself and ‘authenticity talk’, duly placing all mentions of a ‘true self’ in quotations marks—the book is, in a variety of ways, remarkably Romantic.^5^ For Hochschild expresses *explicit* concerns about the problems of self-estrangement, alienation, instrumentalization, and standardization, which she fears have become common features of postindustrial capitalism. Needless to say, this firmly grounds her critique in Romanticism, and its sacred valuation of the sanctity of subjectivity. But what is most striking, at least from the vantage point of today—when explicit endorsements of Romanticism have become thoroughly symbolically polluted—is that *she does not seek to hide this*. Indeed, it is for this reason that we refer to this first mode of appropriation as *explicit Romanticism*.

*The Managed Heart* introduced social scientists to the concepts of ‘feeling rules’, ‘emotional labor’, and ‘deep acting’, thus providing generative analytic resources for making sense of late modern social life. Yet in addition to these contributions to empirical analysis, Hochschild's classic has a clear critical aim: to raise an alarm about the pernicious effects that postindustrial capitalism has on our *inner* lives. Hochschild makes the case that when emotion management is expanded from the private sphere of domestic relations—where each of us accepts to follow feeling rules (the social rules that govern our emotional lives) in order to maintain our personal relations—to the sphere of capitalist work—where feeling rules are dictated by the profit motive—deep pathologies and dysfunctions follow. And it is here, in this *normative* mode, that Hochschild's debts to Romanticism become apparent.

For instance, invoking the early (Romantic) Marx, for whom the evil of capitalism derives from its instrumentalization of the worker, Hochschild remarks, ‘If we can become alienated from goods in a goods-producing society, we can become alienated from service in a service-producing society’ (1983: 7). In her view, because a service economy instrumentalizes not only workers’ bodies, but also their ‘face and feelings’ (ibid.: 136), it threatens our humanity in profound ways. Attending trainings offered to flight attendants, she found that corporate airlines colonize and control their employees’ emotional lives. That is: flight attendants are required, as a part of their job, to mine their emotional capacities by engaging in deep acting, a form of emotion work that entails a conscious engendering of certain feeling states. As a result, their emotional lives are not only ‘stretched into standardized social forms’ (ibid.: 13), but for Hochschild basically cease to be their own. Such an extension of ‘emotion work’ from a ‘private act’ to a ‘public act’ results in a paralyzing estrangement from one's own feelings. Hochschild thus concludes: ‘There is a cost to emotion work: it affects the degree to which we listen to feeling and sometimes our very capacity to feel’ (ibid.: 21).

Thus, according to Hochschild, the emotional labor demanded of flight attendants threatens both their authenticity and their autonomy. Further, these tribulations are merely one specific instance of a more general ‘trend toward standardization and commercialization of emotive offerings’ (1983: 160). ‘Estrangement from display, from feeling, and from what feelings can tell us’ have in her view become ‘ordinary’ (ibid.: 190). At the heart of Hochschild's critique of emotional work in the modern service sector, then, lies a Romantic valuation of authentic subjectivity, and the sacrality of natural (non-coerced) feelings. Over and over again throughout *The Managed Heart*, Hochschild grieves the way corporations have colonized what she calls the ‘private territories of self’ (ibid.: 99). ‘What was once a private act of emotion management is sold now as labor in public-contact jobs’ (ibid: 186). The private emotional sphere ceases to be ‘a sanctuary from abuses of the profit motive’ (ibid.: 161): the most intimate and sacred parts of our selves have become ‘a form of capital’ (ibid.: 185).

In the closing chapter of the book, titled ‘The Search for Authenticity’, Hochschild observes that many of the anxieties she raises were first articulated by none other than proto-Romantic Jean-Jacques Rousseau. She even writes, ‘If Rousseau could sign on as a flight attendant for Delta Airlines in the second half of the twentieth century, he would doubly be interested in learning just *whose* capital a worker's feelings are and just *who* is putting this capital to work’ (Hochschild, 1983: 185; emphasis in original). Yet Hochschild, astute social scientist that she is, does not go so far as to explicitly endorse the Rousseauian line. She avoids explicitly endorsing the (ostensibly sociologically naïve) belief that ‘underneath’ our many layers of social and cultural conditioning there resides such a thing as a ‘natural’ or ‘authentic’ self. Instead, she strategically takes a step back, pointing out as a matter of fact that ‘the value placed on authentic or ‘natural’ feeling has increased dramatically’ (ibid.: 190) to then proceed to speculate on why this might have happened. Yet it is here, in her *diagnosis of the problem*, that her Romanticism shines through. For rather than viewing this development as a naturalization of bourgeois discourse or a capitalist ruse, Hochschild suggests that ‘the value placed on authentic or “natural” feeling has increased dramatically with the full emergence of its opposite—the managed heart’ (ibid.: 190). In other words: ‘The more the heart is managed, the more we value the unmanaged heart’ (ibid.: 192).

Hochschild's *The Managed Heart* features an *explicit Romanticism*, not only because it is firmly rooted in the Romantic episteme, but because it does not shy away from explicitly employing the rhetoric and diagnostic of a Romantic expressive individualism that assumes that the self—the natural, authentic self—precedes the socio-cultural order rather than being shaped by it. Indeed, the book wears its Romanticism on its sleeve. And it is likely for this reason that this style of Romantic social science has become increasingly rare in the ensuing years.

## Explicit Romanticism II, social: Peter Fleming's *Authenticity and the Cultural Politics of Work*

Hochschild's book, written in the US and published in the early 1980s, aimed to map the character of work in the postindustrial service sector and its consequences for the self. Peter Fleming's *Authenticity and the Cultural Politics of Work* was published nearly three decades later in the UK, with social scientists confronting a quite different set of empirical realities, which made them acutely aware of the Romantic dimensions of 21st-century capitalism. Extending ‘radical deconstruction’ of authenticity begun in the 1970s, and in contrast to Hochschild's book, Fleming's is principally concerned with the consequences of the infiltration of Romantic discourse and ideals in the workplace—or what he calls the ‘corporate turn to personal authenticity’ (2009: ix). Over hundreds of pages, he subjects the Romantic thematic to critical scrutiny. Yet for all his bellicosity, Fleming does not desire to rid us of this cultural tradition.

*Authenticity and the Cultural Politics of Work* is first and foremost a form of ideology critique, set on demystifying the regressive consequences of the Romantic turn at work. In Fleming's view, the recent Romantic calls within the corporate workplace to ‘just be yourself’ manufacture an ‘insidious “individualized conformism”’ (2009: 8). Rather than offering ‘real’ freedom and self-expression, ‘corporatized authenticity’ instead provides a mere ‘mimesis or simulation’ of it (ibid.: 27). Whereas Hochschild addresses the distinctly alienating character of commodified emotion work in the service sector, Fleming's critique is thus far more sweeping: in his view, capitalist work *itself* is the problem. He nonetheless shares many of Hochschild's Romantic concerns. What particularly concerns him is that the capitalist embrace of Romanticism ‘enlists the once private dimensions of the individual as a corporate resource’ (ibid.: 31). Thus Fleming, too, takes seriously the threats the new economy and its insidious commercialization and colonization of the lifeworld of workers pose to their authentic selfhood. ‘Authenticity [in its corporate form]’, he writes, ‘is a scaffold upon which the worker can situate a truth of themselves in a forest of illusions and cultural fabrications designed to enlist their most intimate emotions for the firm’ (ibid.: 136). In fact, the normative thrusts of Hochschild's and Fleming's critiques alike rest on the Romantic notion that capitalism is alienating and dehumanizing, with Fleming even going so far as to claim that a ‘systematic lack of life’ (ibid.: 5) characterizes the capitalist workplace.^6^

Yet Hochschild's and Fleming's understandings of authenticity are not identical. Hochschild, as we have seen, espouses an (implicit) expressive individualist Romantic ideal of authenticity, which presupposes the existence of a deeply private and ultimately *pre-social* inner self.^7^ Fleming, by contrast, espouses an ‘imminently social’ (2009: 50) ideal of authenticity. Drawing on the work of post-Marxist thinkers Hardt and Negri (1994), he locates authenticity in ‘the commons’, that is, the lifeworld beyond the productive sphere (Fleming, 2009: 11). In this understanding, capitalism is itself replete with ‘deadening and antisocial structures’ (ibid.: 54), yet parasitically co-opts forces of life, vitality, and authenticity from this noncapitalist realm. To speak of authenticity, then, is for Fleming to speak of an ideal that ‘flows of communal experience’ (ibid.: 149) and that is as such fundamentally *social*.

In so doing, Fleming revives as much as reformulates the tradition of Critical Marxism. For not only does he consider capitalism's alienating character the root of its evil, but underwriting his social diagnoses and prescriptions is nothing less than that distinctly Romantic longing for ‘authentic community’ that combines a fictionalized idea of the past and a utopian future (Löwy and Sayre, 2002: 42). Consider: for Fleming, capitalism in the 21st century has parasitically ravaged ‘the commons’, which implies that there was once an age when our relations and activities were not commodified. The solution is therefore clear: we must liberate the commons from the clutches of capital. Hence, Fleming contends that genuine authenticity will be achieved only once ‘the commons moves out from the shadow of the corporation’ (Fleming, 2009: 165). Despite his ongoing skepticism toward Romanticism in its corporatized form, then, Fleming does not seek to do away with Romanticism. On the contrary: his critique of the Romantic turn at work itself appropriates a Romantic infrastructure, more specifically one reinterpreted to cohere with post-Marxist thought. So, what we find in *Authenticity and the Cultural Politics of Work* is an *explicit Romanticism* like Hochschild's. Yet it differs from hers in that it replaces the expressive individualist notion of a pre-social self that precedes the socio-cultural order with a *social Romanticism* that has been made compatible with social scientific thought.

## Implicit Romanticism: Ofer Sharone's *Flawed System/Flawed Self*

The last of our three books is also the most recent: Ofer Sharone's *Flawed System/Flawed Self*, published in 2013, critically examines cultures of job seeking in America and Israel. Based on interviews and fieldwork with white-collar job seekers, Sharone finds that Romantic discourse has made deeper inroads in processes of job seeking in the US than in Israel. Extending Hochschild's analysis in *The Managed Heart*, Sharone argues that what one finds in the US is a ‘chemistry game’ that requires job seekers to engage in deep acting in order to establish an authentic interpersonal connection with those looking to hire them. In other words, owing to the new spirit of capitalism, American companies increasingly hire on the basis of ‘interpersonal chemistry’ (Sharone, 2013: 64), that is, whether the prospective employee seems like a good ‘fit’ (ibid.: 56). Sharon refers to the emotional labor this demands of American job seekers as ‘self-subjectification’, as it requires ‘tapping into and externalizing elements of one's subjective inner self’ (ibid.: 37). In other words, prospective employees must now train themselves to cultivate the requisite emotions demanded by the prospective employer. They must learn to discipline themselves to *genuinely feel* the way companies want them to.

Despite his stated debts to Hochschild, Sharone's account differs from hers in two key respects. First, Sharone disagrees that the value placed on authenticity is a response to its growing scarcity. Instead, he views it as an instrumental response to the demands of 21st-century capitalism. That is, in Sharone's view, white-collar workers are obsessed with authenticity today *because they have to be*: ‘Workers are turning to the self-help industry not to counteract the effects of emotional labor but, on the contrary, to *perfect* ways of engaging in the emotional labor they perceive to be required by the white-collar labor market—producing and projecting the kind of authentic inner self required by the chemistry game’ (Sharone, 2013: 50; emphasis in original). Sharone thus explicitly rejects the Romantic diagnoses that Hochschild (and Fleming) endorse: that the increased desire for authenticity in the world of work is the result of a lack of ‘genuine’ authenticity. Second, and just as importantly, his account differs in *style*: Sharone writes in a much less personal and committed, more analytically detached, tone than either Hochschild or Fleming.

In fact, Sharone presents himself as a more or less neutral observer, although one who clearly hopes that their empirical analysis will motivate some form of institutional change. Gone are the personal allusions and asides that one finds with Hochschild, and nowhere does one find the denunciatory tone that characterizes Fleming's book. Instead, the implicit expectation of Sharone's book is that the empirical analysis will speak for itself, and that the reader should be left to make up their own minds. However, it is precisely here—between the lines, as it were—that (implicit) Romanticism finds room to breathe. For although Sharone avoids explicit talk of ‘alienation’ or ‘estrangement’, beneath his analytic descriptions lies an unstated set of Romantic concerns and ideals.

As captured in the title of the book, one of the most concerning consequences of the chemistry game, for Sharone, is the way it leads job seekers to engage in ‘highly personalized self-blame’ (2013: 71): it encourages them to view not just their actions, but their *selves* as flawed. This occurs precisely, Sharone explains, because in forcing ‘job seekers to bring to the fore and externalize inner elements of the self’ (ibid.: 170), the chemistry game makes their inner selves the target of scrutiny and assessment. This leaves white-collar American job seekers ‘exposed’ and ‘vulnerable’ to ‘fundamental and debilitating doubt’ about their selves (ibid.: 71). Instead of ‘focusing on one's inadequacy as a job seeker’ job seekers focus on their ‘inadequacy as a person’ (ibid.: 71).

At this point, it warrants asking: *so what?* Please do not misunderstand us: in asking this question, we are not voicing our own view. Rather, we are simply bringing attention to the fact that there is nothing about Sharone's descriptive account that *inherently* warrants condemnation. In fact, one could just as well find this consequence of the chemistry game praiseworthy (indeed, many conservatives would say so). Yet it is apparent that Sharone thinks otherwise. Indeed, in a rare break from scholarly detachment, Sharone refers to the development he describes as ‘insidious’ (2013: 171). So we must ask again: what makes it so? Or, put another way: what are the normative sources from which Sharone draws?

Although Sharone is nowhere explicit about these normative sources, his descriptive empirical analysis has a marked normative appeal and persuasiveness that, we suggest, derives from a Romanticism that remains *implicit*. And what is more, we would contend that *Sharone likely knows this*. In other words, the intellectually detached, neutral tone with which he advances his empirical analysis retains its *critical character*, because Sharone knows he can count on a social scientific readership that *shares his Romantic commitments* to the ‘subjectivity of the individual, the development of the richness of the human personality, in its full affective depth and complexity, but also in the full freedom of its imaginary’ (Löwy and Sayre, 2002: 25). Indeed, like any good social scientist Sharone knows full well that a mere description of how the chemistry game leads the inner self to become ‘vulnerable’ and ‘exposed’, leading job seekers to engage in ‘self-blame’, will activate the moral sensibilities of his (sociologist) readership, for it violates their (our) Romantic conviction that the inner self is a source of infinite value. In fine, what makes self-subjectification and self-blame so ‘insidious’ is precisely the fact that they make the inner self—the site of the Romantic sacred—a site not only of deformity and inauthenticity, but of denigration and devaluation.

Consider Sharone's account of the pitfalls of the chemistry game.^8^ Although he does not use these terms, self-subjectification clearly necessitates a form of self-estrangement and inauthenticity, as job seekers are required to ‘suppress their insecurity’ and project an attitude of ‘utter confidence’ and ‘constant cheerfulness’ (Sharone, 2013: 63). What is more, as Sharone repeatedly emphasizes, this is not simply a matter of surface acting, but deep acting—they must ‘genuinely feel it’ (ibid.: 38). Thus, we might say the chemistry game requires that job seekers alienate themselves from their own true feelings and manipulate their emotional lives for the sake of their prospective employers. Of course, Sharone himself never says so, but we would contend that he is implicitly (and rightly) assuming a Romantic readership, who will interpret his empirical analysis in this way. For such a readership, self-subjectification as described by Sharone cannot but be interpreted as usurping the emotional autonomy of job seekers, and violently forcing a high degree of self-estrangement.

Given the increasingly polluted status of Romanticism in contemporary social science—at least at the level of explicit culture—it is not hard to understand why Sharone avoids the explicit romanticisms of Hochschild and Fleming. Due to his book's implicit appeal to Romantic sensibilities and ideals, however, his descriptive account retains a marked critical thrust and moral persuasiveness. This is why we consider Sharone's analysis an example of *implicit Romanticism*. Eschewing explicit Romantic rhetoric, and presenting itself as a disengaged empirical analysis, a normative infrastructure that is deeply indebted to Romanticism nonetheless gives it normative and existential power.

## The enduring politics of authenticity

In *The Practices of the Self*, Larmore (2010: 53) writes, ‘The attraction that the notion of authenticity holds seems universal and inexhaustible.’ We would agree. But why should this be? What is it about Romanticism and the politics of authenticity it sponsors that leads it to endure in social thought? We propose five reasons.

First, as Gouldner understood well, Romantic social science, in all its forms, stresses the subjective dimensions of good social scientific practice (or perhaps more accurately, the dimensions of social scientific practice that involve subjectivity)—intuition, empathy, insight, and imagination. Accordingly, Romanticism remains a significant strand animating interpretivist, hermeneutic, and anti-naturalist approaches to social science (Watts, 2023), which stress the *sui generis* character of human life, while sharing a suspicion of rationalist, procedural, or positivist approaches. It is therefore not unreasonable to conclude that wherever interpretive social science subsists, so too will Romanticism.

Second, and somewhat paradoxically, Romanticism endures in social thought precisely because of its diffusion within the wider culture. In other words, despite its corporatization, Romantic ideals remain a potent normative source immanent in Western social life. As Taylor (1989) has documented, alongside Enlightenment rationalism, Romanticism has played a formative role in shaping the modern identity, thus it should come as no surprise that it retains its appeal—even among skeptical social scientists. For those interested in changing the world must appeal to normative sources already immanent within society (Walzer, 1983). Indeed, much social and cultural critique reflects an attempt to encourage members of a community to live up to the ideals they already espouse. It is as such quite understandable that much social analysis continues to draw on Romanticism, even when the target of critique is Romanticism's contemporary social manifestations. The relationship between social science and the wider culture is inevitably dialogical, so that good social scientists are aware of the normative and existential assumptions of their readers.

Third, we must not forget what Gouldner called the ‘emancipatory standpoint’ presupposed by Romanticism. As its most basic, the politics of authenticity emerges out of the gap between *subjective experience* and *social understanding*—the gap between our *inner selves* and what *society forces us to be*. In this way, as Rousseau makes so vivid, Romanticism perennially opens the door to radical criticism—by pinpointing the barriers to authenticity it paves the way for critique. The question at the center of Romanticism can be summed up, ‘How much freedom do the members of any state or society have *to be the individuals they are*—how far, in other words, is human authenticity allowed to unfold?’ (Berman, 1972: 22). Accordingly, there is no limit to the demands it can potentially inspire.

Fourth, the language of Romanticism offers an axiological framework—what Taylor (1989), following Gadamer, refers to as a ‘horizon of significance’—that defies the commodifying, instrumentalizing, and rationalizing forces of modernity. In fact, according to Löwy and Sayre (2002: 251), Romanticism reflects *the* critique of modernity:

The Romantic critics have touched … on what was the unthought of bourgeois thought; they have seen what was outside the scope of the liberal individualist worldview: reification, quantification, the loss of qualitative human and cultural values, the solitude of individuals, uprootedness, alienation through merchandise, the uncontrollable dynamic of machines and technology, temporality reduced to the instantaneous, the degradation of nature.

If Löwy and Sayre are correct, then Boltanski and Chiapello's (2005) assumption that the social (socialist) critique of capitalism is more important than its artistic (Romantic) counterpart ultimately belies reality. For without the *qualitative* dimensions captured by the latter, the former arguably loses much of its normative power—in other words, it is reduced to a mere concern with quantification (equality), shorn of a substantive conception of why this matters (authenticity).

Lastly, although no less significantly, Romanticism provides a plausible theodicy in a secular age. The Romantic quest for self-realization—for the sense that one is finally *at home* in the world—offers many in late modernity, critics and non-critics alike, an attractive route to personal salvation. It does so by providing a teleological purpose, imbuing life with transcendent meaning, and staving off the threat of disenchantment. Indeed, it even serves, for some, to revive God in a godless world (Watts and Houtman, 2023b). And with this observed, we wish to suggest that Romanticism has a distinct appeal to social scientists. The reason for this is perhaps counterintuitive, but no less true for being so: because of their sensitivity to the structural and constraining aspects of human life, we believe social scientists often surreptitiously espouse a Romanticism that preserves space for human agency and creativity, beneath the world of social forms.^9^

## Conclusion

Despite its deconstruction, Romanticism continues to serve as an implicit resource for social analysis. Of course, this is not to suggest that *all* social science, or social critique, is Romantic. By no means. But we have focused on the marked tendency of social scientists to defy their own deconstructive critiques in order to draw from the well of Romanticism because, like Gouldner, we believe social scientists should reflexively interrogate their theoretical and normative presuppositions, along with the cultural sources from which they spawn. And also like Gouldner, we believe in the ‘special value’ of the Romantic thematic. Thus, while Romanticism continues to serve as a potent resource for social scientists, it is noteworthy that this cultural tradition is permitted to live on only in increasingly subtle and subterranean forms. This raises the question of whether, in the coming years, we shall gradually witness its dissolution. Perhaps we are reaching a point in history where Romanticism's social scientific relevance and potential has been exhausted? Admittedly, if this is the case, we cannot help but feel some regret. For, despite its limitations and deficiencies, to rid ourselves of this structure of thought and feeling would be to rid ourselves of not just what many of us hold dear, but also ‘one of the most politically explosive human impulses’ (Berman, 1972: xix).

## Notes

We would like to thank Chris Renwick and the rest of the editorial team at *History of the Human Sciences* for smoothly shepherding this article through the review process.

1. In fact, Gouldner (1980: 46) says plainly, ‘Critical Marxism is romantic.’

2. Breines (1977: 474) refers to Hegel as ‘a most Romantic critic of Romanticism’.

3. This strand of critical theory continues to be carried forward in the present day by Harmut Rosa (2018). Rosa’s theory of resonance, indebted the work of Charles Taylor, unabashedly draws upon Romantic infrastructure. This might lead some to question whether Romanticism remains as symbolically polluted in social science as we profess. In response, we would contend that Rosa is the exception that proves the rule.

4. As Campbell (1987) noted some time ago, Romanticism has long been central to consumer culture. Thus it is this cultural tradition’s infiltration into the sphere of work that distinguishes the late modern era, for this marks a historic break from what Weber (2005[1904/5]) called the ‘inner-worldly asceticism’ that has long reigned in the workplaces of Western capitalist societies (see Bell, 1976).

5. Given that this may be interpreted as a criticism, we wish to clarify that it should not be. Although it is true that *Romantic* is often used as a term of abuse (especially in academic contexts), we use it in a purely descriptive sense.

6. With this in mind, it is worth noting that, according to Abrams (1973: 431), the ‘ground-concept’ for Romantics is ‘life’: ‘Life is itself the highest good, the residence and measure of other goods, and the generator of the controlling categories of Romantic thought.’

7. This Romantic ideal of authenticity shines through when we examine Hochschild’s concern about the ‘altruistic self’ that society socializes women to adopt. For Hochschild, this social role puts women at a disadvantage, because it risks undermining their autonomy and leading them to lose touch with themselves (see Hochschild, 1983: 196).

8. Importantly, Sharone never in fact refers to these *as* pitfalls. But this is precisely our point. He does not need to, for in presenting them in the way he does, there is an assumption that he will active our (shared) normative frameworks, and thus the critical thrust of his analysis will become apparent.

 9. Indeed, it is precisely for this reason that Weber was a Romantic (Watts and Houtman, 2023b).
